# Correlation between low-level viremia and hepatitis B-related hepatocellular carcinoma and recurrence: a retrospective study

**DOI:** 10.1186/s12885-021-08483-3

**Published:** 2021-10-14

**Authors:** Furong Sun, Zhifei Liu, Bingyuan Wang

**Affiliations:** 1 Department of Elderly Gastroenterology, The First Hospital of China Medical University, Shenyang, 110001 China; 2 School of Pharmacy, China Medical University, Shenyang, 110001 China

**Keywords:** Chronic hepatitis B, Hepatocellular carcinoma, Low-level viremia, Alpha-fetoprotein, Virologic response

## Abstract

**Background:**

Low-level viremia generally refers to detectable HBV DNA levels lower than 2000 IU/mL. Studies show that low-level viremia is a risk factor for hepatocellular carcinoma. The aim of this study was to explore the characteristics of low-level viremia patients with hepatitis B-related hepatocellular carcinoma and identify prognostic factors after curative hepatectomy.

**Methods:**

Data from chronic hepatitis B patients with hepatocellular carcinoma receiving curative hepatectomy for the first time in the first hospital of China Medical University were studied. Patients were divided into two groups based on preoperative HBV DNA levels: group 1 (low-level viremia group, HBV DNA < 2000 IU/mL) and group 2 (HBV DNA ≥ 2000 IU/mL).

**Results:**

Of the 212 patients, 104 patients were in group 1 and 108 patients were in group 2. There was a lower proportion of patients with HBsAg levels > 250 IU/mL (the upper limit of detection in our laboratory) in group 1 than in group 2 (71.2% vs. 86.1%, *P* < 0.01). The percentage of patients with a tumor diameter < 5 cm was 67.3% in group 1 and 37.0% in group 2 (*P* < 0.000). The percentage of tumor recurrence was 40.4% (42) in group 1 and 54.6% (59) in group 2 (*P* < 0.05). Median recurrence-free survival was 30.1 months in group 1 and 17.6 months in group 2 (*P* < 0.01). Multivariate analysis showed that a tumor diameter ≥ 5 cm (hazard ratio [HR] = 1.819, 95% confidence interval [CI] 1.193–2.775, *P* = 0.005), intrahepatic metastasis (HR = 1.916, 95% CI 1.077–3.407, *P* = 0.027), and an HBV DNA level ≥ 100 IU/mL (the lower limit of detection in our laboratory, HR = 2.943, 95% CI 1.916–4.520, *P* < 0.000) were independent prognostic factors associated with an increased risk of hepatocellular carcinoma recurrence.

**Conclusion:**

Preoperative low-level viremia was related with a long tumor recurrence interval and complete virologic response after curative hepatectomy was associated with a lower risk of hepatocellular carcinoma recurrence.

## Background

Hepatitis B viral (HBV) infection remains a serious global public health problem. HBV infection is the leading cause of hepatocellular carcinoma (HCC) worldwide, accounting for 33% of cases [1]. In China, chronic hepatitis B (CHB) contributes to approximately 84% of HCC [2]. The incidence of HBV-related deaths due to liver cirrhosis and/or HCC dramatically increased between 1990 and 2013 [3], resulting in a large health and economic burden. Therefore, effective control of CHB is crucial. Serum HBV DNA level is essential for informing decision-making regarding antiviral treatment and the subsequent monitoring of disease progression. A prospective cohort study showed that an HBV DNA level more than 10,000 copies/mL was a strong predictor of HCC development [4]. Long-term, maintained virologic response (VR) is associated with a lack of progression, even in those with decompensated liver cirrhosis [5]. Therefore, an important goal of antiviral treatment is to obtain VR.

Low-level viremia (LLV) can be detected in CHB patients including those taking antiviral therapy, generally referring to detectable HBV DNA levels lower than 2000 IU/mL. Recently, an increasing number of studies have shown that LLV plays a crucial role in the diagnosis and prognosis of HCC. It has been reported that LLV is associated with a higher risk of HCC and poorer overall survival, compared with those who maintained VR [6, 7]. LLV patients with a relatively low viral load can still benefit from effective antiviral therapy, showing as significantly decreased risk of HCC [7]. More importantly, antiviral therapy significantly reduced HCC recurrence after R0 hepatic resection [8].

Although the exact incidence of LLV is not known, the potential LLV population is likely considerable. The timing of antiviral therapy for LLV is controversial. Generally, LLV without liver cirrhosis or elevated ALT is considered inactive CHB, and is not an indication for antiviral therapy [2, 9]. However, due to the risk for HCC and potential benefits of reduced risk of HCC recurrence, it may be necessary for LLV patients to receive antiviral therapy at an earlier CHB stage and to maintain a long-term VR with undetectable HBV DNA loads. Continuous monitoring of cirrhosis and HCC is also important during routine follow-up.

Characteristics of CHB have been well described, and studies have revealed common characteristics of LLV. However, an overall understanding of LLV is lacking. Thus, there is still an urgent need to describe LLV characteristics and distinguish LLV patients from other CHB patients. This would help inform suitable management strategies for LLV patients and improve early detection of HCC. In this study, we explored the characteristics of LLV in HCC patients who had undergone curative hepatectomy. Then, prognostic factors were further analyzed in an effort to understand the differential characteristics of LLV patients versus those with high-level viral loads.

## Methods

### Study population

We obtained data on CHB patients who had been diagnosed with malignant liver tumors and underwent curative partial hepatectomy by open or laparoscopic hepatectomy in the first hospital of China Medical University between 2011 and 2018. Diagnosis of HCC was based on two types of imaging examinations including liver ultrasound, computed tomography, and magnetic resonance imaging. Diagnosis was further confirmed via analysis of resected specimens.

**Fig. 1:**
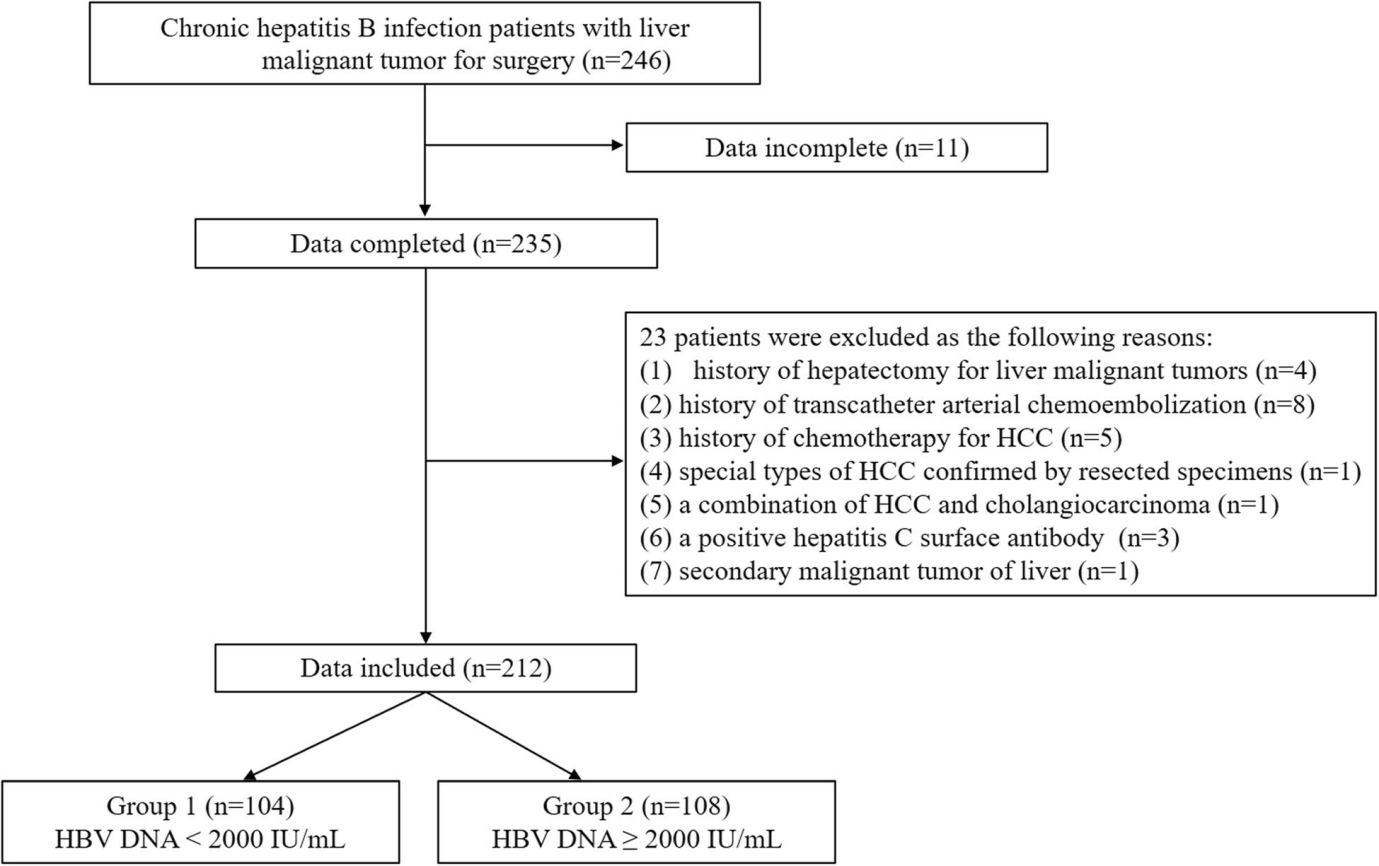
Flowchart of patients. HCC: hepatocellular carcinoma

Inclusion criteria included: (1) first time hepatectomy for HCC, (2) history of CHB with positive hepatitis B surface antigen (HBsAg), (3) no extrahepatic metastasis, and (4) above 18 years old. Exclusion criteria included: (1) data incomplete, (2) history of hepatectomy for liver malignant tumors, (3) history of transcatheter arterial chemoembolization for HCC, (4) history of chemotherapy for HCC, (5) special types of HCC confirmed by resected specimens, (6) a combination of HCC and cholangiocarcinoma, (7) positive hepatitis C surface antibody with increased HCV-RNA loads, (8) secondary malignant tumor of liver. Detailed patient information is shown in Fig. 1. This study was approved and the need for informed consent was waived by the institutional review board of the First Hospital of China Medical University.

### Preoperative data

All patients were divided into two groups based on their preoperative serum HBV DNA levels: Group 1 included patients with serum HBV DNA levels less than 2000 IU/mL, Group 2 included patients with serum HBV DNA levels ≥2000 IU/mL.

Medical records were collected and included sex, age, history of antiviral therapy, smoking, and alcohol consumption. An alcohol consumption less than 30 g/d for males and 20 g/d for females was defined as moderate alcohol consumption; and an alcohol consumption more than 60 g on one occasion was defined as heavy episodic drinking [10]. Characteristics of liver tumors were carefully recorded, including the number of nodules, the maximum diameter, differentiation, capsule formation, intrahepatic metastasis, satellite nodules, and portal vein tumor thrombosis.

Blood samples were taken after an overnight fasting. Parameters including leukocyte count, hemoglobin, platelet count, alanine aminotransferase (ALT), aspartate aminotransferase (AST), γ-glutamyl transpeptidase (GGT), alkaline phosphatase (ALP), serum albumin (ALB), prothrombin time (PT), international normalized ratio (INR), creatinine, alpha-fetoprotein (AFP), HBsAg (upper limit of detection was 250 IU/mL in our laboratory), serum HBV DNA (lower limit of detection was 100 IU/mL in our laboratory), and hepatitis B e antigen (HBeAg) were then evaluated. Data from preoperative ultrasound, computed tomography, and/or magnetic resonance imaging were also collected.

### Follow-up and assessment of recurrence

All patients were followed-up once every month or once every 3 months at the outpatient department. Patients with detectable HBV DNA or liver cirrhosis were given antiviral therapy pre- and post-operation. Complete VR was defined as the HBV DNA levels persistently lower than 100 IU/mL. Partial VR was defined as the HBV DNA load decreased by 2 or more log values when compared with the basic viral load. Virological breakthrough was defined as the HBV DNA loads increased by 1 or more log values when compared with the lowest viral load either during the antiviral treatment or redetected during follow-up. Blood samples were taken after an overnight fasting for analysis of liver function, AFP, and HBV DNA levels at every visit. Liver ultrasound, computed tomography, and/or magnetic resonance imaging were taken at the same time to monitor for new lesions in the liver.

Tumor recurrence was defined as (1) new lesions in the liver suspected by liver ultrasound and furthered confirmed by computed tomography or magnetic resonance imaging with or without elevated serum AFP; (2) new lesions in the liver detected by liver ultrasound, computed tomography, or magnetic resonance imaging, and further confirmed in resected specimens.

### Statistical analysis

Continuous variables are presented as mean ± standard deviation or median (interquartile range). Quantitative variables were compared by Student’s *t* test for continuous variables with a normal distribution or the Mann-Whitney nonparametric *U* test. Categorical variables were analyzed by Chi-square test and are expressed as numbers and percentages. Recurrence-free survival was calculated by the Kaplan-Meier method and the differences were compared by log-rank test. Univariate and multivariate analyses were performed by the Cox proportional hazards regression model with stepwise selection of variables. Statistical analysis was performed using IBM SPSS statistics software version 22.0 (IBM, Armonk, NY, USA). A *p* value < 0.05 was considered statistically significant.

## Results

### Characteristics of patients

A total of 169 males and 43 females were enrolled in the study; 104 patients were in group 1 and 108 patients were in group 2. The mean age was 54.5 ± 9.6 years, ranging from 23 to 81 years old. The percentage of patients with a history of smoking or alcohol consumption was 18.4 and 14.2%, respectively. There were no significant differences in sex, age, history of smoking, and alcohol consumption between the two groups (*P* > 0.05). Details are shown in Table 1.

**Table 1:** Characteristics of patients and tumors, preoperative laboratory tests, and follow-up

	Total	Group 1	Group 2	*P*
	(*n* = 212)	(*n* = 104)	(*n* = 108)	
**Characteristics of patients**				
Age (years)	54.5 ± 9.6	55.2 ± 10.0	53.9 ± 9.2	> 0.05
Sex (male, %)	169 (79.7)	84 (80.8)	85 (78.7)	> 0.05
Smoking (yes, %)	39 (18.4)	17 (16.3)	22 (20.4)	> 0.05
Drinking (yes, %)				
No drinking	182 (85.8)	91 (87.5)	91 (84.3)	> 0.05
Moderate drinking	21 (9.9)	9 (8.7)	12 (11.1)	
Heavy episodic drinking	9 (4.2)	4 (3.8)	5 (4.6)	
HBsAg (> 250 IU/mL, %)	167 (78.8)	74 (71.2)	93 (86.1)	< 0.01
Positive HBeAg (n, %)	61 (28.8)	18 (17.3)	43 (39.8)	< 0.000
**Preoperative laboratory tests**				
Leukocyte (10^9^/L)	5.41 ± 1.83	5.44 ± 1.81	5.38 ± 1.86	> 0.05
Hemoglobin (g/L)	145.0 ± 18.8	145.9 ± 19.2	144.1 ± 18.5	> 0.05
Platelet (10^9^/L)	160.4 ± 70.1	166.8 ± 68.5	154.3 ± 71.4	> 0.05
ALB (g/L)	40.2 ± 3.9	41.5 ± 3.5	38.8 ± 3.7	< 0.000
ALT (U/L)	31 (22 - 46)	25 (18 - 33)	40 (28 - 54)	< 0.000
AST (U/L)	32 (24 - 46)	26 (20 - 32)	40 (31 - 55)	< 0.000
ALP (U/L)	92.4 ± 47.4	85.1 ± 43.1	99.5 ± 50.5	0.02
GGT (U/L)	51 (32 - 98)	37 (23 - 60)	74 (44 - 123)	< 0.000
PT (s)	13.9 ± 0.9	13.8 ± 1.0	13.9 ± 0.9	> 0.05
INR	1.08 ± 0.08	1.07 ± 0.09	1.08 ± 0.08	> 0.05
Creatinine (μmol/L)	67.44 ± 32.75	71.94 ± 44.76	63.1 ± 12.15	< 0.05
AFP (ng/mL)	69.57 (6.38 - 1210.00)	23.09 (3.65 - 706.3)	145.50(11.25 - 1210.00)	< 0.01
AFP < 10 ng/mL (n, %)	65 (30.7)	41 (39.4)	24 (22.2)	< 0.01
**Characteristics of tumors**				
Differentiation				
Well (%)	39 (19.3)	22 (22.2)	17 (16.5)	> 0.05
Moderate (%)	130 (64.4)	59 (59.6)	71 (68.9)	
Poor (%)	33 (16.3)	18 (18.2)	15 (14.6)	
Cirrhosis	167 (78.8)	78 (75.0)	89 (82.4)	> 0.05
Diameter (< 5cm, %)	110 (51.9)	70 (67.3)	40 (37.0)	< 0.000
Metastasis in the liver (yes, %)	24 (11.3)	10 (9.6)	14 (13)	> 0.05
Capsule formation (yes, %)	130 (61.3)	55 (52.9)	75 (69.4)	< 0.05
**Follow-up**				
Tumor recurrence (n, %)	101 (47.6)	42 (40.4)	59 (54.6)	< 0.05
AFP (ng/mL)	5.86 (2.77 - 195.68)	3.40 (2.18 -37.77)	16.12 (3.68 -671.05)	< 0.000
AFP < 10 ng/mL (n, %)	120 (57.1)	71 (68.9)	49 (45.8)	< 0.000

*ALB* serum albumin, *ALT* alanine aminotransferase, *AST* aspartate aminotransferase, *ALP* alkaline phosphatase, *GGT* γ-glutamyl transpeptidase, *PT* prothrombin time, *INR* international normalized ratio, *AFP* alpha-fetoprotein

A total of 167 (78.8%) patients demonstrated a serum HBsAg level > 250 IU/mL, with a lower percentage in group 1 than in group 2 (71.2% vs. 86.1%, *P* < 0.01). There were 28.8% patients with positive HBeAg, with a higher percentage in group 2 than in group 1 (39.8% vs. 17.3%, *P* < 0.000). Details are shown in Table 1.

### Antiviral treatment

Most patients had records of antiviral treatment. A total of 196 patients (92.5%) accepted pre- and post-operative antiviral treatment. Entecavir was administered to 181 patients (85.4%). Other antiviral agents included lamivudine (five patients), adefovir dipivoxil with or without entecavir (five patients), tenofovir fumarate diofuroxil (two patients), telbivudine (two patients), and interferon α (one patient). Complete VR was observed in 46 patients and 80 patients, pre- and post-operation, respectively, in group 1. Complete VR and partial VR were observed in 48 patients and 52 patients, pre- and post-operation, respectively, in group 2. More patients in group 1 showed post-operative virological breakthrough than in group 2 (11 vs. 3). Details are presented in Table 2.

**Table 2:** Antiviral treatment and virological response

	Total	Group 1	Group 2
	(*n* = 212)	(*n* = 104)	(*n* = 108)
Antiviral treatment, n (%)	196 (92.5)	95 (91.3)	101 (93.5)
data not available, n (%)	16 (7.5)	9 (8.7)	7 (6.5)
Preoperative complete VR, n (%)	46 (21.7)	46 (44.2)	-
Post-operative complete VR, n (%)	128 (60.4)	80 (76.9)	48 (44.4)
Post-operative partial VR, n (%)	53 (25.0)	1 (1.0)	52 (48.1)
Post-operative virological breakthrough, n (%)	14 (6.6)	11 (10.6)	3 (2.8)

*VR* virological response

### Characteristics of tumors

Liver cirrhosis was observed in most enrolled CHB patients, with a percentage of 75.0% in group 1 and 82.4% in group 2 (*P* > 0.05). The percentage of well, moderate, and poor differentiation was 19.3% (39), 64.4% (130), and 16.3% (33), respectively (*P* > 0.05). Capsule was observed in 61.3% (130) of cases, the percentage of tumors with capsule formation was 52.9% in group 1 and 69.4% in group 2 (*P* < 0.05). The percentage of patients with a tumor diameter less than 5 cm was 67.3% in group 1 and 37.0% in group 2 (*P* < 0.000). Details are shown in Table 1.

### Preoperative laboratory tests

Routine blood tests showed that the mean leukocyte count, platelet count, and hemoglobin were in the normal ranges with no significant difference between groups (*P* > 0.05). Similarly, the mean prothrombin time and international normalized ratio was normal (*P* > 0.05). Mean ALP and median ALT, AST, and GGT were higher in group 2 than in group 1 (*P* = 0.02 for ALP, *P* < 0.000 for ALT, AST, and GGT). The mean albumin was lower in group 2 than in group 1 (38.8 ± 3.7 vs. 41.5 ± 3.5, *P* < 0.000). Median serum AFP concentration was 69.57 ng/mL for all patients; a much higher median AFP level was observed in patients in group 2 (145.50 vs. 23.09, *P* < 0.01). A total of 41 (39.4%) patients in group 1 and 24 (22.2%) patients in group 2 had an AFP level under 10 ng/mL (*P* < 0.01). Details are shown in Table 1.

### Follow-up

**Fig. 2:**
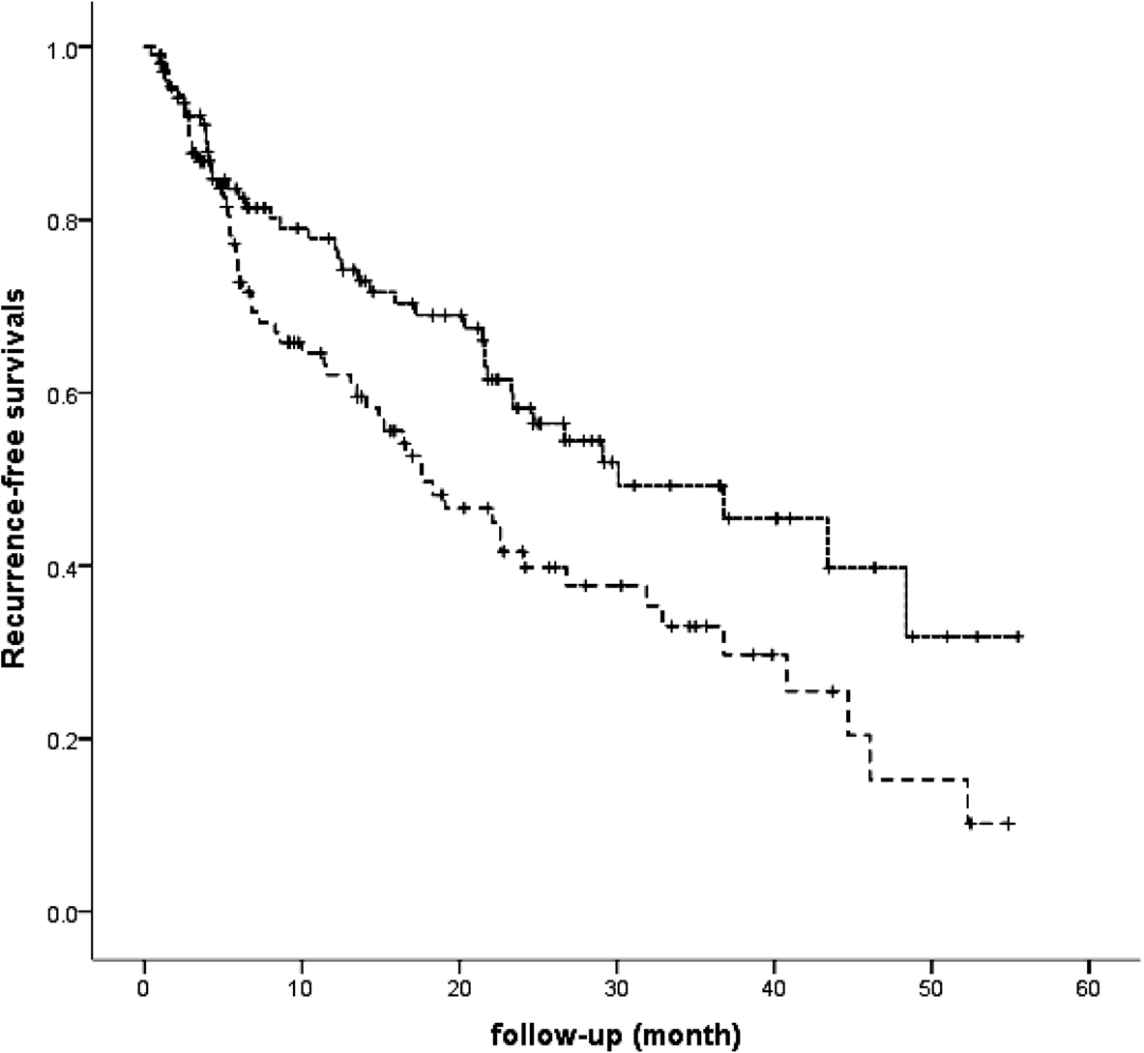
Comparison of tumor recurrence-free survival between the 2 groups. Kaplan-Meier curve of tumor recurrence-free survival based on preoperative HBV DNA levels. The median tumor recurrence-free survival was 30.1 months in group 1 and 17.6 months in group 2 (*P* < 0.01) (log-rank *P* = 0.015)

The median follow-up was 13.5 months. At the end of follow-up, a total of 101 (47.6%) patients experienced HCC recurrence, 42 (40.4%) patients in group 1 and 59 (54.6%) patients in group 2 (*P* < 0.05) (Table 1). Median recurrence-free survival was 30.1 months in group 1 and 17.6 months in group 2 (log-rank *P* = 0.015, *P* < 0.01) (Fig. 2). A total of 128 patients obtained complete VR during follow-up, exhibiting lower recurrence rate (36.7% vs. 64.3%) and longer recurrence-free survival (40.8 months vs. 11.4 months, *P* < 0.000) compared with patients without complete VR. In patients with tumor recurrence, the percentage of AFP levels < 10 ng/mL was 35.7% (15/42) and 27.1% (16/59) in group 1 and group 2, respectively.

Parameters significantly associated with hepatocellular recurrence on univariate analysis were then used for multivariate analysis. Analysis showed that a tumor diameter ≥ 5 cm (hazard ratio [HR] = 1.819, 95% confidence interval [CI] 1.193–2.775, *P* = 0.005), intrahepatic metastasis (HR = 1.916, 95% CI 1.077–3.407, *P* = 0.027), and an HBV DNA level ≥ 100 IU/mL (HR = 2.943, 95% CI 1.916–4.520, *P* < 0.000) were independent prognostic factors associated with an increased risk of HCC recurrence. Details are shown in Table 3.

**Table 3:** Univariate and multivariate analyses for recurrence of hepatocellular carcinoma

Factors	No. Patients	Univariate Analysis		Multivariate Analysis	
		HR (95% CI)	*P*	HR (95% CI)	*P*
Sex					
Male	169 (79.7%)	0.850 (0.509 - 1.419)	0.533		
Female	43 (20.3%)				
HBV DNA level preoperation					
< 2000 IU/mL	104 (49.1%)	1.629 (1.095 - 2.423)	0.016	1.007 (0.653 - 1.553)	0.976
≥ 2000 IU/mL	108 (50.9%)				
Cirrhosis					
Yes	167 (78.8%)	0.812 (0.513 - 1.286)	0.375		
No	45 (21.2%)				
Tumor diameter					
< 5cm	110 (51.9%)	1.934 (1.300 - 2.877)	0.001	1.819 (1.193 - 2.775)	0.005
≥ 5cm	102 (48.1%)				
Capsule formation					
Yes	130 (61.3%)	0.850 (0.573 - 1.261)	0.42		
No	82 (38.7%)				
Metastasis in the liver					
Yes	24 (11.3%)	2.077 (1.196 - 3.607)	0.009	1.916 (1.077 - 3.407)	0.027
No	188 (88.7%)				
AFP preoperation					
< 10 ng/mL	65 (30.7%)	1.714 (1.089 - 2.698)	0.02	1.462 (0.919 - 2.324)	0.109
≥ 10 ng/mL	147 (69.3%)				
HBsAg					
< 250 IU/mL	45 (21.2%)	1.042 (0.644 - 1.686)	0.867		
≥ 250 IU/mL	167 (78.8%)				
HBeAg					
Positive	61 (28.8%)	1.162 (0.765 - 1.764)	0.481		
Negative	151 (71.2%)				
HBV DNA level at follow-up					
< 100 IU/mL	128 (60.4%)	2.725 (1.836 - 4.044)	< 0.000	2.943 (1.916 - 4.520)	< 0.000
≥ 100 IU/mL	84 (39.6%)				

Data showed that a tumor diameter ≥ 5 cm (HR = 1.819, 95% CI 1.193–2.775, *P* = 0.005), intrahepatic metastasis (HR = 1.916, 95% CI 1.077–3.407, *P* = 0.027), and an HBV DNA level > 100 IU/mL (HR = 2.943, 95% CI 1.916–4.520, *P* < 0.000) were independent prognostic factors for HCC recurrence. *HR* hazard ratio, *CI*, confidence interval

## Discussion

In this cohort of CHB patients with curative hepatectomy, most LLV patients achieved complete VR during follow-up and experienced longer recurrence-free survivals. Post-operative serum HBV DNA levels > 100 IU/mL were an independent prognostic factor for HCC recurrence after curative hepatectomy, confirming that complete VR after curative hepatectomy could dramatically improve tumor recurrence-free survivals. Though multivariate analysis showed a lower risk of HCC recurrence in those with post-operative serum HBV DNA levels < 100 IU/mL, there was still a risk of fibrosis progression and HCC [6, 11]. There were 11 patients with preoperative LLV showed virological breakthrough during follow-up. Among them, post-operative HBV DNA loads ≥2000 IU/mL were observed in seven patients (four patients without any record of antiviral treatment), with shorter tumor recurrence-free survivals (less than 6 months in five patients). These results indicate that LLV without effective antiviral treatment is a risk factor for HCC recurrence. Effective antiviral treatment and complete VR are important for LLV patients.

Serum HBsAg levels are an important characteristic of CHB and useful for identifying stage of disease and effects of nucleos(t)ide analogues therapy [12, 13]. More importantly, HBsAg levels are associated with the risk of progression to HCC, especially in LLV patients with negative HBeAg [14]. In this cohort, a total of 167 patients showed serum HBsAg concentrations > 250 IU/mL, possibly reflecting high amount and enhanced transcriptional activity of covalently closed circular DNA inside the hepatocytes [15]. As we know, HBsAg declines very slowly in the most cases through the nature course, even HBV DNA has been undetectable [16]. Therefore, serum HBsAg levels could be an important indicator for monitoring antiviral treatment in those with LLV or even undetectable HBV DNA. Initial therapeutic target may be at a level of 100 IU/mL, because serum HBsAg levels less than 100 IU/mL suggested a high probability of spontaneous HBsAg clearance [15]. Unfortunately, we could not detect the exact quantitative HBsAg levels in these patients, limited to the test method in our laboratory. Further studies are needed to explore the role of serum HBsAg on CHB control and even in HCC development.

The main difference in tumor characteristics between the two groups was tumor diameter, an important prognostic factor in HCC patients [17]. Multivariate analysis verified that a tumor diameter ≥ 5 cm and intrahepatic metastasis were two independent prognostic factors for recurrence of HCC after curative hepatectomy. However, the cases of intrahepatic metastasis were not high in this cohort, probably due to the curative hepatectomy. Thus, tumor diameter seems a much more important characteristic among LLV patients in this cohort. As we showed, there were 67.3% LLV patients with tumor diameter < 5 cm, which might be related with a lower tumor recurrence rate. However, more studies are needed to confirm this. Besides, small tumor size may be related with less serious liver injury, showing as low-level liver enzymes in LLV patients.

The usage of AFP in diagnosing HCC is controversial. A cutoff value of 20 ng/mL shows high sensitivity but low specificity, whereas a high cutoff value at 200 ng/mL lowers sensitivity dramatically [18], leading to a non-negligible risk of misdiagnosis. Dynamic changes of AFP levels could also be observed in CHB patients, showing as elevated AFP levels before antiviral therapy and decreased AFP levels after antiviral therapy in patients with active CHB [19]. In this study, we used a lower cutoff value of 10 ng/mL and found the proportions of pre- and post-operative AFP levels < 10 ng/mL were high in both groups. More importantly, in patients with tumor recurrence, there were 35.7% (15/42) patients in group 1 and 27.1% (16/59) patients in group 2 showed AFP levels < 10 ng/mL, respectively. These results suggest that low AFP levels are not safe enough on excluding HCC and tumor recurrence, especially in LLV patients. The effectiveness of AFP at 10 ng/mL on screening HCC should be more rigorous evaluated in the future.

There were some limitations in this study. First, metabolism-related fatty liver disease is gradually becoming an important risk factor for HCC. Data on body weight and height were missing in some patients, therefore body mass index could not be calculated and compared between groups. Second, the follow-up time was not long enough in some patients. All these factors should be further studied.

## Conclusions

LLV patients with HCC were characterized by smaller tumor size, less serious liver injury, and lower AFP levels compared to patients with HBV DNA ≥ 2000 IU/mL. Preoperative LLV was related with a long tumor recurrence interval, and complete VR after curative hepatectomy was associated with a lower risk of hepatocellular carcinoma recurrence. A more suitable management strategy should be established to reduce the incidence of HCC in LLV patients. HBsAg levels could be an important indicator for monitoring the effects of antiviral therapy in patients with LLV or even undetectable HBV DNA.

## Data Availability

The datasets used and/or analysed during the current study are available from the corresponding author on reasonable request.

## Abbreviations

ALT: Alanine aminotransferase
ALP: Alkaline phosphatase
AFP: Alpha-fetoprotein
AST: Aspartate aminotransferase
CHB: Chronic hepatitis B
CI: Confidence interval
GGT: γ-glutamyl transpeptidase
HR: Hazard ratio
HBeAg: Hepatitis B e antigen
HBsAg: Hepatitis B surface antigen
HBV: Hepatitis B viral
HCC: Hepatocellular carcinoma
LLV: Low-level viremia
VR: Virologic response
